# Supplementary material for: A fast and effective detection framework for whole-slide histopathology image analysis
Source: PLoS One. 2021 May 12;16(5):e0251521. doi: 10.1371/journal.pone.0251521 (PMC8115773; doi:10.1371/journal.pone.0251521)
Supplement: S1 Appendix — (DOCX) [file pone.0251521.s001.docx]

A fast and effective detection framework for Whole-Slide Histopathology Image analysis

Jun Ruan^1^, Zhikui Zhu^1^, Chenchen Wu^1^, Guanglu Ye^1^, Jingfan Zhou^1^, Junqiu Yue^2*^

Appendices

**Supplementary 1: Testing results of transfer-learning**

Due to the different sizes of the input image for different networks, we resized image blocks to default shape, then the features of patches were extracted by these pre-trained transfer-learning models. And we replaced the original top layer with a new one to connect each feature extraction part, which consists of a Global Average Pool (GAP) and two fully-connected layers. We used the prepared patches on three different magnifications to fine-tuning the top layer of each transfer model and tested the accuracy of these 18 models. All testing results of transfer-learning are shown in S1 Table.

S1 Table Accuracy of patches classification on different magnification using different Transfer models

| Model name | Parameters  (M) | Image size | Binary Accuracy | | |
| --- | --- | --- | --- | --- | --- |
|  |  |  | 10× | 20× | 40× |
| Inception_v3 | 23.9 | 224x224x3 | 0.9231 | 0.9573 | 0.8835 |
| **DenseNet121** | 8.1 | 299x299x3 | 0.9329 | 0.9640 | 0.8946 |
| **DenseNet169** | 14.3 | 299x299x3 | 0.9273 | 0.9656 | 0.8978 |
| **DenseNet201** | 20.2 | 299x299x3 | 0.9329 | 0.9651 | 0.8946 |
| ResNet50 | 25.6 | 299x299x3 | 0.8604 | 0.9300 | 0.8146 |
| Inception_ResNet_v2 | 55.9 | 224x224x3 | 0.9218 | 0.9599 | 0.8904 |
| VGG16 | 138.4 | 299x299x3 | 0.9186 | 0.9537 | 0.8757 |
| MobileNet_v2 | 3.5 | 299x299x3 | 0.9063 | 0.9506 | 0.8752 |
| NASNet(mobile) | 5.3 | 299x299x3 | 0.9100 | 0.9595 | 0.8849 |

According to the results of transfer-learning, DenseNet has the best performance of feature extraction for pathological image blocks. Moreover, the patches under 20× magnification have the best distinguishable characteristics which can be extracted by CNN, as a result of the balance of texture details and texture range in view. Here we use the same image size under different magnifications, and the center of the patches under different magnifications are coincident. Although the 10× patches have a larger field of view, they are downsampled to the same size resulting in the loss of texture and degradation of classification performance. Compared to 20× magnification, the classifiers under 10× are a little worse. Under 40× magnification, the field of view of the patches becomes very small, and many of them are extracted from the transitional zone from tumor to normal. Thus, the texture features extracted from such 40× patches are not significant and typical. So, it is difficult to train a better classifier under this magnification. On the other hand, under the same image size, the prediction under higher magnification can more accurately represent the tumor probability at the sampling point (the center of patches).

**Supplementary 2: Our adaptive sampling algorithm**

We extracted the thumbnail $I$ of a WSI $X$ under 1.25× (Level 5) and separated it using the SLIC [30] algorithm. The boundaries B of the segmented superpixel regions $S$ were extracted. Then, we performed regular sampling at equally spaced intervals on the boundaries of $S$. Here, the number of superpixels $S$ is proportional to the area of the WSI. The area of each superpixel $S$ is approximately 1000 pixels under 1.25×, which is equivalent to the area of four 256×256 patches under 20×. In this way, a set of center coordinates of patches is obtained and used to generate the first gradient map in the feature space.

**Algorithm 1:** **Regular sampling** **based on superpixels**

**Input:**

$X$: WSI

$A$: area of each superpixel

$d$: spaced intervals of sampling

Superpixel regions $S$ ← SLIC segmentation (*X*, *A*)

Boundaries B ← find boundaries (S)

Coordinates of the sampling center $C_{R}$ ← regular sampling (B, d)

**Return** S, $C_{R}$

In our algorithm, iterative adaptive sampling was followed by regular sampling. Our iterative strategy was divided into two stages overall. The first stage was the adaptive sampling within a full WSI. The second stage was the adaptive sampling within the enabled superpixels in the WSI. We will discuss these stages in detail below.

**Algorithm 2: Sampling point generation algorithm**

**Input:**

$N_{A}$: number of sample points extracted

$W_{img}$, $H_{img}$: width and height of WSI under 1.25×

$M_{grad}$: feature gradient map

$f_{grad}$: heuristic factor on $M_{grad}$

$T_{grad}$: threshold of $f_{grad}$

$S_{enable}$: enabled superpixel set

If $f_{grad}$ < $T_{grad}$:

$C^{k}$← pseudorandom sampling (${2N}_{A}$, $W_{img}$, $H_{img}$)

$\mathcal{G}^{k}$← $\left( g_{i}^{k} | \max_{c_{j}^{k}} \left( grad(c_{j}^{k}, M_{grad}) | \left\| c_{j}^{k}-c_{i}^{k} \right\|<\varepsilon\right) \right)$

$\acute{C}^{k}$← select top based on gradient ($N_{A}, C^{k},\mathcal{G}^{k}$)

**Return** $\acute{C}^{k}$

Else If in the first stage $f_{grad}\geq T_{grad}$:

$C^{k}$← pseudorandom sampling (${4N}_{A}$, $W_{img}$, $H_{img}$)

$\acute{C}^{k}$←$\left( c_{i}^{k} | grad(c_{i}^{k},M_{grad})\geq f_{grad} \right)$

If length ($\acute{C}^{k}$) < $N_{A}$:

new $C^{k}$←pseudorandom sampling within neighborhood (${4N}_{A}$, $\acute{C}^{k}$)

$\ddot{C}^{k}$←$\left( c_{i}^{k} | grad(c_{i}^{k},M_{grad})\geq f_{grad} \right)$

$C^{k}$← select randomly ($N_{A}, \acute{C}^{k}\cup\ddot{C}^{k}$)

**Return** $C^{k}$

Else If in the second stage $f_{grad}\geq T_{grad}$:

$C^{k}$← pseudorandom sampling (${4N}_{A}$, $S_{enable}$)

$\acute{C}^{k}$←$\left( c_{i}^{k} | grad(c_{i}^{k},M_{grad})\geq f_{grad} \right)$

**Return** $\acute{C}^{k}$

Here, $C^{k}$ is the coordinate set of sampling points at the $k$th iteration. $c_{i}^{k}$ is the $i$th coordinate in $C^{k}$. The function $grad(c_{i}^{k},M_{grad})$ means obtaining the gradient $g_{i}^{k}$ at $c_{i}^{k}$ under $M_{grad}$. $\varepsilon$ indicates the size of the neighborhood, which defaults to a 16×16 square under 1.25×.

**Algorithm 3: Adaptive gradient-based sampling**

**Input:**

$\mathbb{M}$: CNN-trained model

$X$: WSI

$T$: maximum iterations

$N_{A}$: number of sample points extracted

$A$: area of each superpixel

$d$: spaced intervals of sampling

$T_{grad}$, $T_{\rho}$, $T_{f}^{sp}$and $T_{g}^{sp}$: thresholds

$M_{grad},f_{grad}\mathcal{, H,\ldots}$← $\phi$

$S$, $C_{R}$ ← regular sampling based on superpixels ($X,A,d$)

$S_{enable}=S$

For $i=1$ to in $T$ do:

$C_{A}$ ← sampling point generation ($N_{A},M_{grad},f_{grad},$

$T_{grad},S_{enable},\ldots$ )

$C$ = $\left\{ \begin{aligned} C_{R}\cup C_{A}, i=1 \\ C_{A}， i>1 \end{aligned} \right.$

Predictions $\mathcal{F}$ ← patch classification ($\mathbb{M}$, $C$)

$M_{feat}$ ← feature map interpolation ($\mathcal{F}$, $C$)

$M_{grad}$ ← feature gradient ($M_{feat}$)

$\mu_{0},\mu_{1}$ ← clustering ($M_{grad}$)

$f_{grad}=\min\left( 0.5*\left( \mu_{0}+\mu_{1} \right),0.3 \right)$

${avg}_{\rho_{dt}}$ ← average local sampling density within the neighborhood ($\mathcal{H,}C$)

$\mathcal{H}$ ← $\left( \left( c_{i},f_{i} \right) | c_{i}\in C,f_{i}\mathcal{=F(}c_{i}) \right)\mathcal{\cup H}$

If ${avg}_{\rho_{dt}}>T_{\rho}$:

$\rho_{sp}$ ← local sampling density within superpixels ($\mathcal{H,}S$)

$S_{enable}$ ← update enabled regions ($S,\rho_{sp},{T_{\rho},T}_{f}^{sp},T_{g}^{sp}$)

If $S_{enable} is \phi$:

**Return** $\mathcal{H}$

When $S_{enable}$ is updated, the adaptive sampling process is executed again until the average of $\rho_{dt}$ reaches $T_{\rho}$. By iteratively updating $S_{enable}$ and sampling, $S_{enable}$ finally becomes an empty set, and the entire algorithm will end. We only returned the coordinates $c_{i}$ and predictions $f_{i}$ (1-dim feature) of each sample point in all iterations.

**Supplementary 3: Our method for lesion-based Evaluation**

For postprocessing, the extraction of the corresponding coordinates is divided into two steps. In the first step, the two new thresholds $T_{p,low}$ and $T_{p,high}$ are calculated to distinguish between tumor and normal with the goal of reducing false positives. Based on the positive patch-level predictions, we used the Isolation Forest algorithm [39] to separate the outliers, and calculated the two centers of the K-means (K = 2) clustering of these outliers. If the distance between the two cluster centers is large enough (> 1.0) in the feature space, then we argued that the larger center in the feature space corresponds to sample points inside the core tumor regions. The smaller center corresponds to the sample points at the edge of the tumor regions. Therefore, let $T_{p,high}$ be the probability of the minimum of the predictions in the core regions, and let $T_{p,low}$ be the probability of the maximum of the predictions in the edge regions. Otherwise, let $T_{p,low}$ and $T_{p,high}$ be 0.5 and the probability of the mean of the two cluster centers.

In the second step, we extracted the coordinates of the local H-maxima based on a tumor probability heatmap. The nonlocal maxima were suppressed within a given distance to ensure that the distance between these extreme points is sufficient. Finally, all coordinates with a tumor probability greater than $T_{p,high}$ are preferentially output, and the coordinates with a tumor probability greater than $T_{p,low}$ are output, sorted by tumor probability until the upper limit of the number of outputs is reached.

**Supplementary 4: More examples**

In S1-S4 Figs, the contour lines of the probability maps are shown in (A). Here the black, red, orange and white contour lines correspond to tumor probabilities of 0.3, 0.5, 0.6, and 0.8, respectively. (B) shows the predictions corresponding to the left side using our adaptive sampling method. The yellow regions in (B) indicate the ground truth.


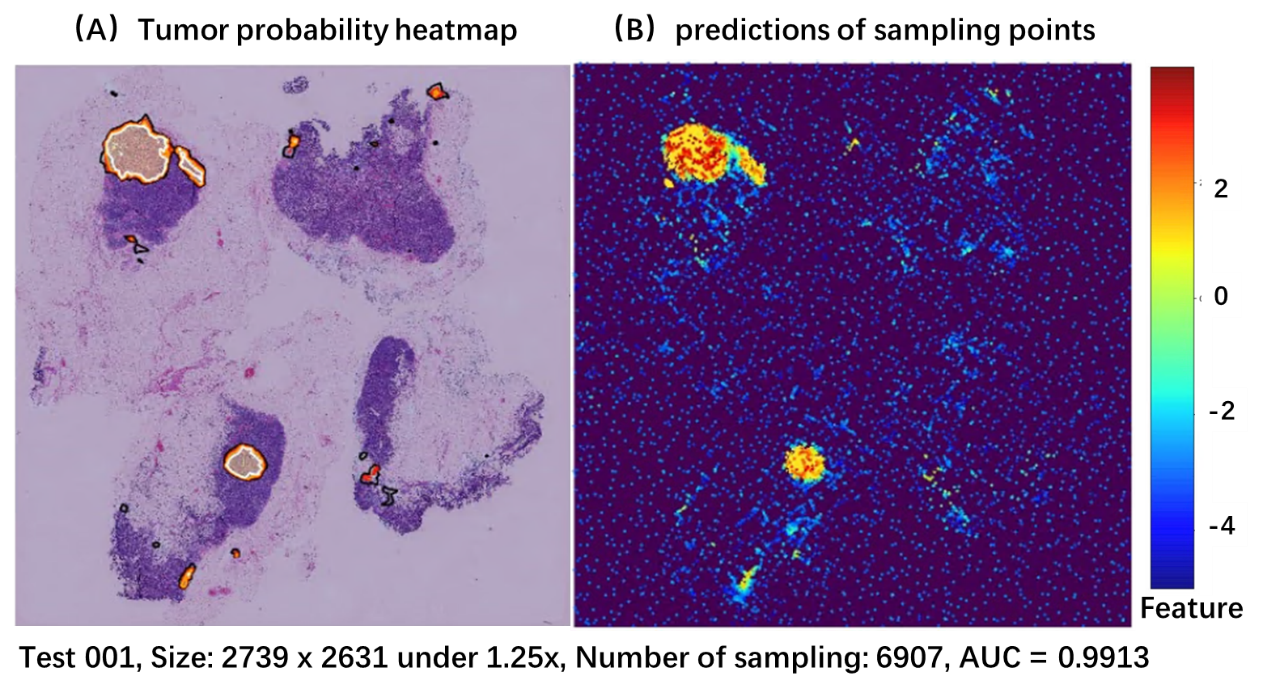


S1 Fig Tumor probability heatmap and predictions on Test 001


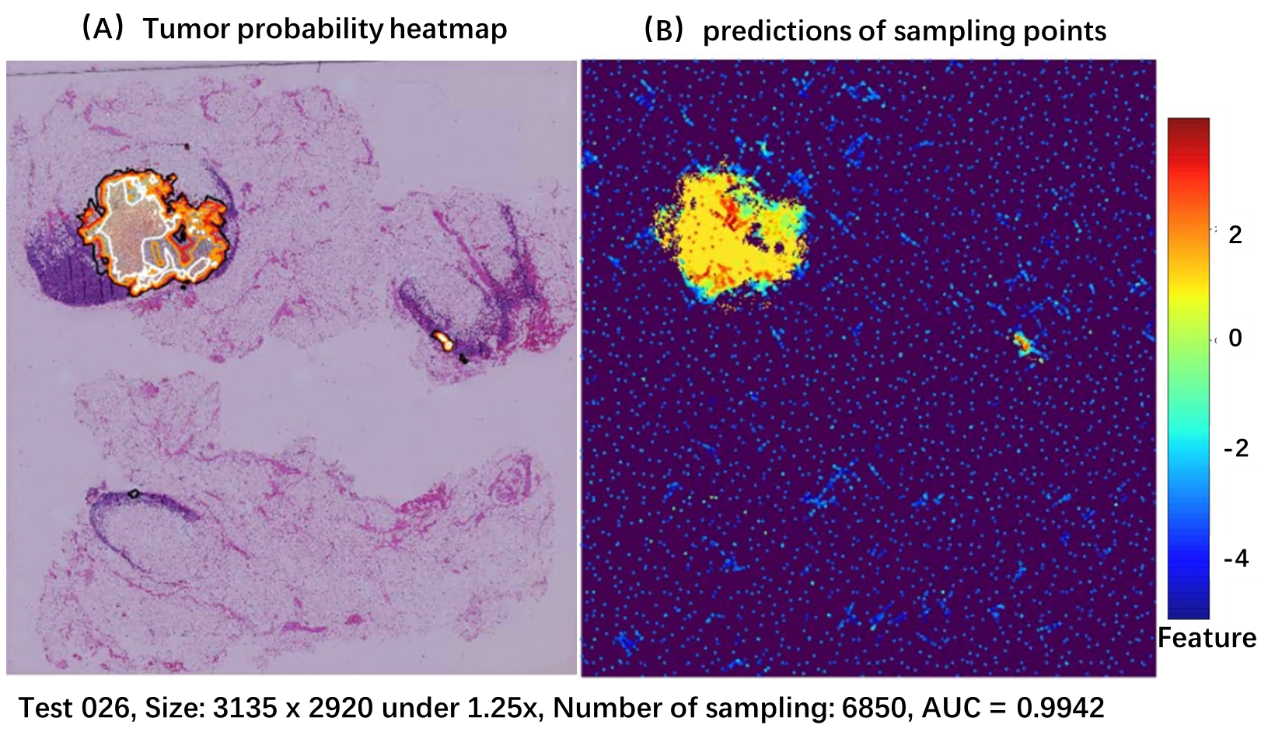


S2 Fig Tumor probability heatmap and predictions on Test 026


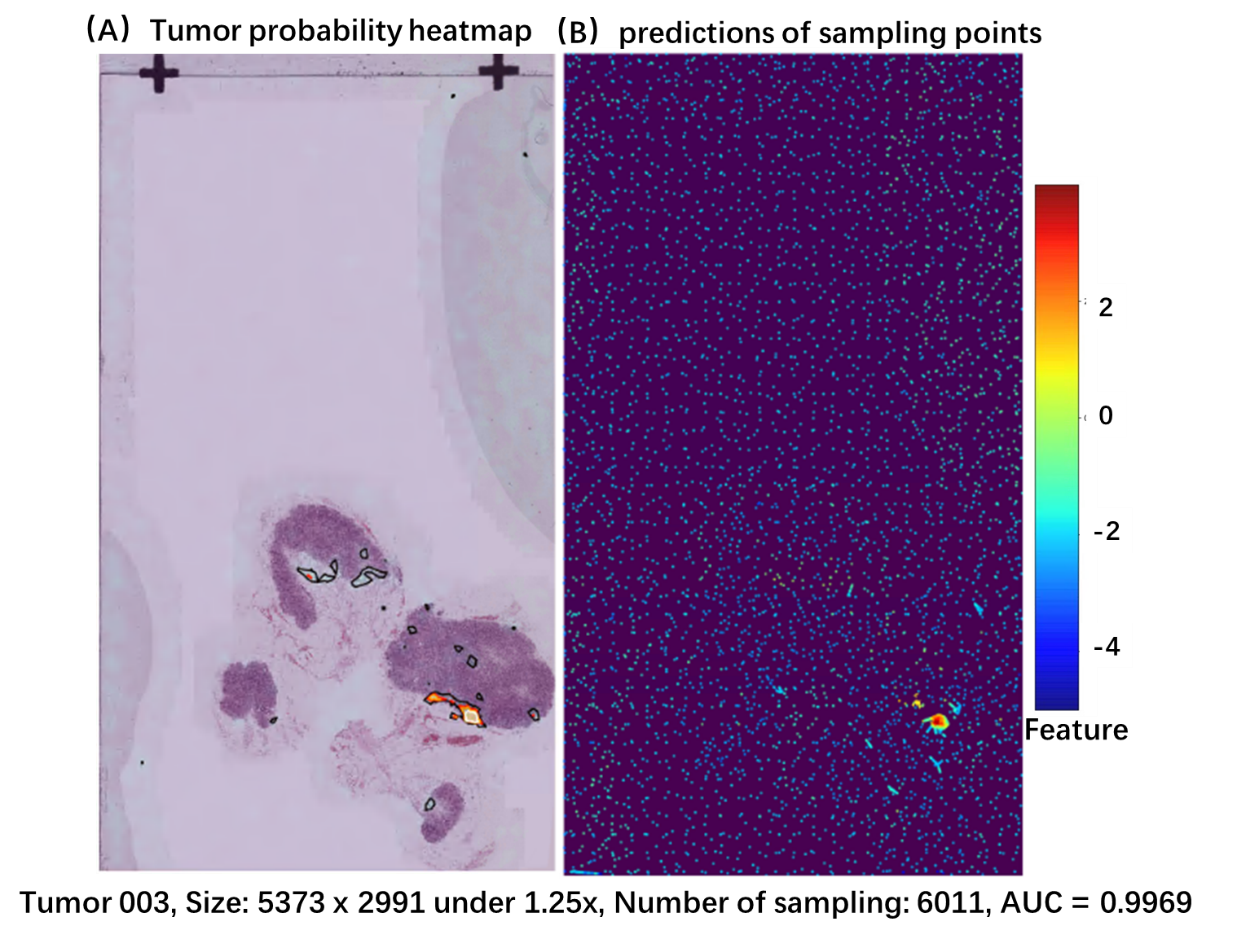


S3 Fig Tumor probability heatmap and predictions on Tumor 003


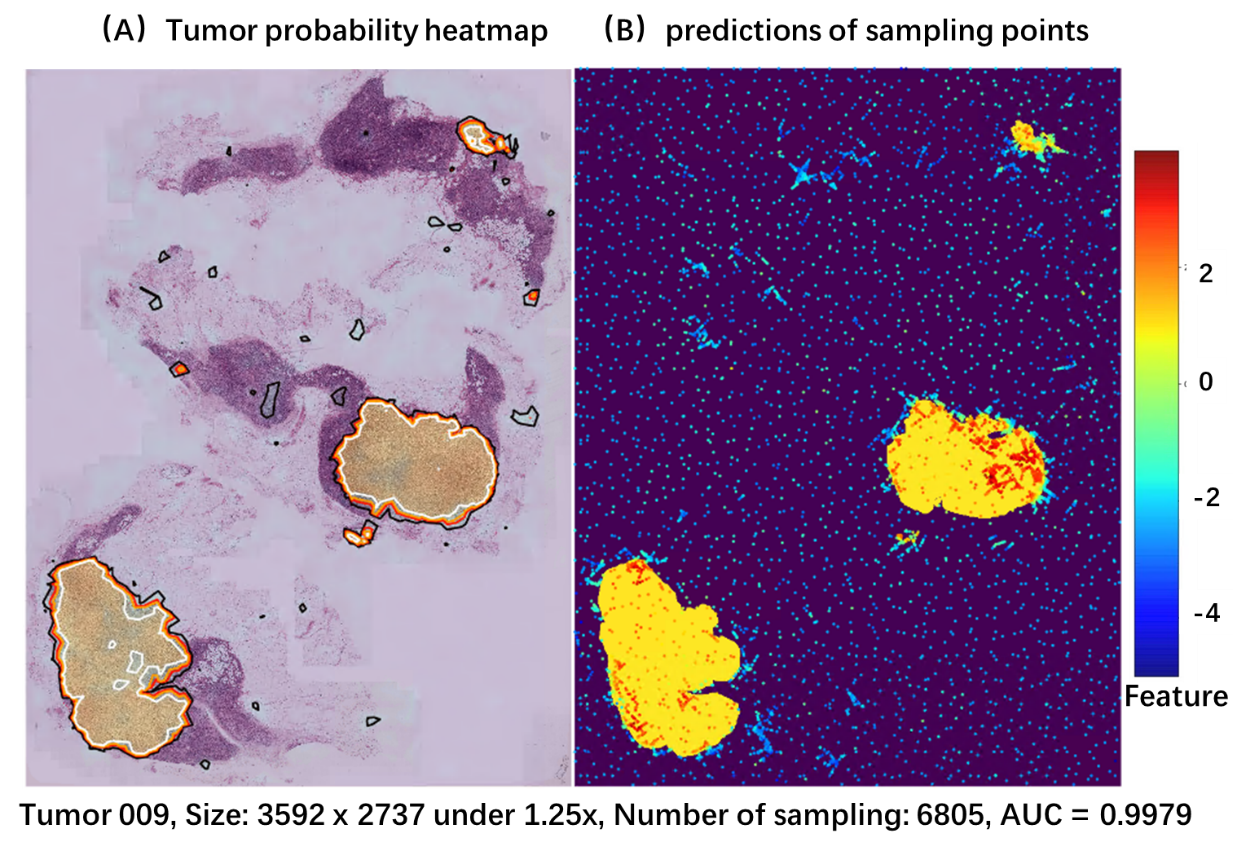


S4 Fig Tumor probability heatmap and predictions on Tumor 009
