# Supplementary material for: A fast and effective detection framework for whole-slide histopathology image analysis
Source: PLoS One. 2021 May 12;16(5):e0251521. doi: 10.1371/journal.pone.0251521 (PMC8115773; doi:10.1371/journal.pone.0251521)
Supplement: Attachment — Submitted filename: PONE-D-21-02087 Response to Reviewers.docx [file pone.0251521.s002.docx]

**Original Manuscript ID: PONE-D-21-02087**

**Original Article Title:** " A fast and effective detection framework for Whole-Slide Histopathology Image analysis."

**To**: PLOS ONE Editor

**Re**: Response to reviewers

Dear Editor,

Thank you for allowing a resubmission of our manuscript, with an opportunity to address the reviewers' comments.

We are uploading (a) our point-by-point response to the comments (below) (response to reviewers), (b) an updated manuscript with yellow highlighting indicating changes, and (c) a clean updated manuscript without highlights.

Best regards,

Junqiu Yue et al.

**Reviewer#1, Concern # 1:** in Line 134, what is the “simple threshold-based segmentation method”?

**Author response:** For excluding the obvious background region, we apply three fixed-level thresholds in HSV to the thumbnail image of WSIs by experience. In the S channel, its value is required to be greater than 0.1. The value of the V channel is required to be between 0.2 and 0.8. These have been introduced in Line 135-136 of the original paper.

**Reviewer#1, Concern # 2:** in Line 155-156, “For the choice of patch-based classifier structure, we chose nine classic pre-trained ImageNet networks to test the patch-based classifier under three magnifications”, what is the motivation to use these methods?

**Author response:** We tried to find a suitable classifier structure using transfer learning. The features of patches were extracted by nine pre-trained models. And we replaced the original top layer with a new one to connect each feature extraction part, which consists of a Global Average Pool (GAP) and two fully-connected layers. We used the prepared patches on three different magnifications to fine-tuning the top layer of each transfer model and tested the accuracy of these 18 models. All testing results of transfer-learning are shown in Supplementary Table 1. According to the results of transfer-learning, the DenseNet family has the best performance of feature extraction for pathological image blocks. Therefore, we construct our network based on DenseNet.

Supplementary Table 1 Accuracy of patch-based classification on different magnification using different Transfer models

| Model name | Parameters  (M) | Image size | Binary Accuracy | | |
| --- | --- | --- | --- | --- | --- |
|  |  |  | 10× | 20× | 40× |
| Inception_v3 | 23.9 | 224x224x3 | 0.9231 | 0.9573 | 0.8835 |
| DenseNet121 | 8.1 | 299x299x3 | **0.9329** | **0.9640** | **0.8946** |
| DenseNet169 | 14.3 | 299x299x3 | **0.9273** | **0.9656** | **0.8978** |
| DenseNet201 | 20.2 | 299x299x3 | **0.9329** | **0.9651** | **0.8946** |
| ResNet50 | 25.6 | 299x299x3 | 0.8604 | 0.9300 | 0.8146 |
| Inception_ResNet_v2 | 55.9 | 224x224x3 | 0.9218 | 0.9599 | 0.8904 |
| VGG16 | 138.4 | 299x299x3 | 0.9186 | 0.9537 | 0.8757 |
| MobileNet_v2 | 3.5 | 299x299x3 | 0.9063 | 0.9506 | 0.8752 |
| NASNet(mobile) | 5.3 | 299x299x3 | 0.9100 | 0.9595 | 0.8849 |

We revised the manuscript accordingly, and the above table has been added to the supplementary materials.

**Reviewer#1, Concern # 3**: in Line 162-163, “Our modified network has only 0.3 million parameters with 3 dense blocks defined in DenseNet, and 163 each block consists of 12 convolution layers. The growth rate (‘k’ in [17]) is 16”, what did authors use Densenet40 ? Due to the normally used structure DenseNet is Densenet41, lease give the structure of Densenet40. Why did the authors set growth rate k=16?

**Author response:** Our network consists of three dense blocks defined in DenseNet. Each block consists of 6 dense layers that each contains two convolution layers. Between two adjacent dense blocks, there is a translation layer that consists of one convolution layer. And only two transport layers are used here. The network also contains a convolutional layer at the input and a fully connected layer at the top. Therefore, we refer to this network architecture as DenseNet40 (=3x6x2+2x1+1+1=40).

Here we set the growth rate *k* to 16 to reduce the parameters of the model by using very narrow layers, at the same time, keeping up with the performance of patch classification.

In the corresponding section of the revised manuscript, we provide a detailed description.

**Reviewer#1, Concern # 4:** in Line 181-199 , these two loss functions are well-known, so the authors do not need to introduce them so detailed.

**Author response:** We very much appreciate the comments. We have trimmed this section.

**Reviewer#1, Concern # 5:** most references are ok, but some are too old and some new papers should be read and added.

[1] Gastric histopathology image segmentation using a hierarchical conditional random field （2020）

[2] RMDL: Recalibrated multi-instance deep learning for whole slide gastric image classification （2019）

[3] Superpixel-Based Conditional Random Fields (SuperCRF): Incorporating Global and Local Context for Enhanced Deep Learning in Melanoma Histopathology （2019）

[4] A review for cervical histopathology image analysis using machine vision approaches (2020)

[5] A Cervical Histopathology Image Clustering Approach Using Graph Based Features (2020)

[6] A Comprehensive Review of Markov Random Field and Conditional Random Field Approaches in Pathology Image Analysis (2021)

[7] Computerized Spermatogenesis Staging (CSS) of Mouse Testis Sections via Quantitative Histomorphological Analysis (2020)

**Author response:** Thank you for the suggestion about these references. We updated the introduction section by explaining the recent studies related to WSI analysis, as suggested by the reviewer. Since the manuscript focuses on the classification and localization of tumor regions at the pixel level on a WSI, we do not cite all the above literature. We hope that the reviewer will be satisfied.

**Reviewer#1, Concern # 6**: Figure 1-6 have low quality to improve. And the layouts of some tables are uncomfortable to read. Please update.

**Author response:** Thank you very much for the feedback about the quality of the figures and tables. We will try our best to improve.

**Reviewer#1, Concern # 7:** There are still many grammar problems, such as Line 29, 30, 31, 35, 41-42, 46, 81-82, 97, 102, 105-107. Except these problems, there are still many others to improve.

**Author response:** We very much appreciate the comments. We tried to revise the paper. We hope the English grammar would be better now. We also hope that we answer the reviewer questions properly.

**Reviewer#2, Concern # 1**: 80 000 x 80 000 at 40x : 2G. I am surprised with the size at 40x I would say 20 G ?

**Author response:** Whole-slide images are stored in a multi-resolution pyramid structure. Image files contain multiple down-sampled versions of the original image. Each image in the pyramid is stored as a series of tiles, to facilitate rapid retrieval of subregions of the image. In other words, the WSIs have been highly compressed. For example, the compressed storage space of the No. 76 tumor sample is 2.24GB with 114688 x 100352 pixels at 40x, but the uncompressed size is 32.2GB.

**Reviewer#2, Concern # 3:** “Because the patches at the edge of annotations are usually transitional regions from tumor tissue to normal tissue, most of the “hard examples” are concentrated in these positions. The same is true for the labeling of patch pairs under double magnifications”

Are you sure ? For me, the border of the tumoral areas are quite visually marked even I agree there is a transition that makes it more interesting to be precise from a computational point of view.

**Author response:** We apologize for the inaccurate description and confusion here. In the latest version, the labeling method has been adjusted. The labeling of a patch is determined by the proportion of the tumor area within the patch. When the proportion of tumor area is less than 50%, this patch is normal (Label 0); otherwise, it is tumor (Label 1). For the labeling under the combination of double magnifications, we adopted the "or" logic here.

Compared with the method of judging whether the center point of a patch is in the ground truth annotation, these updated labels make the patch-based classifier more robust. The accuracy of the predicted segmentation boundary is attempted to be ensured by our adaptive algorithm, which can perform further sampling in suspicious regions or boundaries.

The experimental results in the manuscript are obtained using the above-mentioned lastest annotation method. In the revised version, we made corresponding corrections.

**Reviewer#2, Concern # 4:** Here, the sampling interval in the normal regions is larger than that in the tumor region.

Here stands for where ? What do you mean ?

**Author response:** Sorry for not expressing clearly here. We balanced the number of positive and negative samples in a WSI by dynamically changing the sampling interval of different regions. If the area of normal regions in a WSI is much larger than the tumor area, we will increase the sampling interval in the normal regions. In this way, the number of negative samples in a WSI does not exceed 5~6 times the number of positive samples. Then, we constructed balanced training sample sets (1:1) through random sampling for improving the performance of the patch-based classifier. We have corrected this part in the manuscript.

**Reviewer#2, Concern # 5:** because the prediction of a patch with the same size under 40× is more accurate for the center of the patch. ??

Rephrase it or explain it more clearly

**Author response:**

We use patches of the same image size under different magnifications, and the centers of the two patches under different magnifications are coincident. According to the experimental results of transfer learning in Supplementary Table 1, we observed the following phenomena.

The patches under 20× have the best distinguishable characteristics which can be extracted by CNN, as a result of the balance of texture details and texture range in view. Although the 10× patches have a larger field of view, they are downsampled to the same size resulting in the loss of texture and degradation of classification performance. Compared to 20×, the classifiers under 10× are a little worse. Under 40×, the field of view in a patch becomes very small in a patch. When the patches are extracted from the transitional zone from tumor to normal near the edge of annotations, these patches under 40× are no significant and typical texture features, and even look the same as the patches in normal regions. So, it is difficult to train a better classifier under this magnification individually. On the other hand, the patch-based classifier calculates a tumor feature based on the entire 256 × 256 image, and the calculated patch-level prediction is stored in a tumor feature map based on the central coordinate of the patch.

Thus, with the same image size, the prediction under higher magnification can more accurately represent the tumor feature (probability) at the sampling point (the center of a patch). From the perspective of spatial location, we argue that the prediction of a patch with the same size under 40× is more accurately express the tumor feature at the center of the patch, and facilitate the generation of more detailed segmentation boundaries. Moreover, in the pixel segmentation experiment, the accuracy under the 20× and 40× magnifications alone is better than that under the 10× alone. In the end, our patch-based classifier chose the combined input under 20× and 40×.

**Reviewer#2, Concern # 6:** The structure of the patch-based classifier was derived from DenseNet-40, so we called it the Double Magnification Combination (DMC) patched-based classifier.

So ?? I do not see the logical implication ?

**Author response:** We apologize for the illogical expression here; this “so” is redundant.

**Reviewer#2, Concern # 7:** The growth rate (‘k’ in [17]) is 16. is set to 16 and explain what it is

**Author response:** The growth rate is a hyper-parameter of the DenseNet model. In a dense block, from input to output, the number of feature-maps of the convolutional layers continues to increase, and the increment is the growth rate mentioned here.

We set the growth rate to 16 to reduce the parameters of the model by using very narrow layers, at the same time, keeping up with the performance of our patch classification.

**Reviewer#2, Concern # 8:** Here, y refers to the ground truth under double magnification, y 20 and y 40 and so forth. The prediction is the same in classif, isn’t it ?

**Author response:** As mentioned earlier, the labeling of a patch is determined by the proportion of the tumor area within the patch. When the proportion of tumor area is less than 50%, this patch is normal (Label 0); otherwise, it is tumor (Label 1). For the labeling under the double magnification combination, we adopted the "or" logic here.

Thus, inside the normal and tumor regions, the labels y20 and y40 of a pair of patches overlap at the center point are the same. The y20 and y40 of a pair of patches extracted from the edges of manual annotations may be different. And the label y takes their logical union.

**Reviewer#2, Concern # 9:** Here, α is 1.0, and w is the weight of the L-GM loss, which is 0.001 by explain alpha et w why 0,5 and w if w is set to a fixed value. It complexity the method for no added value. Or you make w varying ?

**Author response:** α is a hyper-parameter of L-GM loss. In the reference, its default value is 1. Here, we followed this setting.

w is the weight of the regularization term of the loss function ${loss}_{2}$, and It is an empirical value.

The first factor of 0.5 in ${loss}_{2}$ indicates that the two cross-entropy losses at both magnifications have the same weight, that is, the classification error is reflected in their average under both magnifications.

**Reviewer#2, Concern # 10:** Ca and Cr a and r stands for what

Globally as there is a lot of parameters make it clear the choice of subscript

**Author response:** We very much appreciate the comments. In the revised manuscript, we tried our best to clarify the meaning of these symbols.

In the pseudo-code of Algorithm 1, $C_{R}$ refers to the set of center coordinates of patches, which are obtained by regular sampling based on superpixel segmentation. The subscript "R" here is the initials of “Regular”. $C_{A}$ refers to the set of center coordinates of patches, which are obtained by random sampling (Quasi-Monte Carlo) process. The subscript "A" here is the second letter of “random”, since "R" has already been used before.

**Reviewer#2, Concern # 11:** Here, T f represents the lower limit of the tumor feature with a superpixel, generally set to -1, which indicates that a superpixel within a tumor probability of more than 27% should be considered

Make it clearer please??

**Author response:** Because the output of our binary patch-based classifier is the tumor feature of a patch, if we use the Sigmoid function to regress a feature into a tumor probability, the feature value -1 corresponds to the tumor probability of 27%. Here the threshold $T_{f}^{sp}$ represents the lower limit of the tumor feature in a superpixel, generally set to -1. When there is a feature larger than $T_{f}^{sp}$ in a superpixel, it means that there is a point with tumor probability greater than 27% inside. In the next iteration, such superpixels will be further explored.

We have revised this in the manuscript.

**Reviewer#2, Concern # 12:** Fig 4 is not very clear ?

the 12 points for instance

**Author response:** We very much appreciate the comments. We will try our best to improve.

**Reviewer#2, Concern # 13:** Here, we did not use fully-conv (FC) net to directly generate a heatmap under 1.25×, because this required higher hardware requirements (GPU memory capacity).

But you use a GPU in the results ? (see “The performance test was performed on a PC with a 3.2 GHz Intel i7-8700 CPU with 16 GB of memory and an NVIDIA GeForce GTX 1080 8 GB”.)

**Author response:** Due to limited resources, we only put the training and prediction of the DMC patch-based classifier to run on GPU. While in our adaptive sampling, the generation of sampling coordinates, the operations of feature maps $M_{feat}$ and gradient maps $M_{grad}$ are all done on the CPU.

**Reviewer#2, Concern # 14:** When the features under both magnifications are used at the same time in DMC, the performance was improved by nearly 2~3%. Because a pair of patches overlap at the center point and the field of view is different, so the spatial attention mechanism was introduced. For the performance of patch-level detection, our experience is the use of L-GM loss in training has no significant effect on single-input or dual-input classifiers.

Results interesting : x20 seems to be enough

Is it very useful to add ‘0x then. ?

**Reviewer#2, Concern # 15:** Although our training and testing sets contain millions of patches extracted from WSIs, the patches extracted during the adaptive sampling process were still more complex.

I do not understand the logical articulation.

**Author response:** These two issues are related and we give clarification here together.

Pathologists usually check images by changing their magnification and scope in the WSI. Ways to use images under a wider range of magnifications are worth studying. Inspired by this, we investigated the patch-based classifier with multiple magnifications. Regarding the F1 scores in Table 2, there is not much difference in accuracy between single-input or dual-input patch-based classifiers, but there is a significant difference in the results of pixel-level segmentation. In the pixel-level segmentation task, the F1 scores of each patch-based classifier are much lower than the scores of the classifier during training and testing. The loss of the pixel-level segmentation task is not used to optimize the performance of patch-based classifiers; it represents the generalization performance of the classifier. This is because, although we extracted millions of patches from WSI for training these patch-based classifiers, the input images during our adaptive random sampling are almost impossible to be the same as those in the training set. In other words, the patches extracted during the adaptive sampling are more varied. Moreover, the prediction error at any sampling point has an impact on the accuracy of the segmentation boundary near it. The superposition effect brought by the sampling mechanism makes it possible to obtain correct results only when the robustness of the patch-based classifier is sufficient. Regarding the F1 score of pixel level, the performance of our adaptive sampling algorithm on DMC is nearly 20% higher than that of the classifiers with single magnification. This shows the advantages of the dual input structure.

**Reviewer#2, Concern # 16:** Table 3 interesting

HCH very different from Camelyon : can you elaborate on it a bit more as you started to do in the following lines.

This is because the area of the tumor regions in each slide of HCH is on average 8 to 9 times larger than Camelyon16, but the number of regions is generally relatively small. In other words, the detection target is relatively significant. Therefore, HASHI is more suitable for the detection of such WSIs, and our proposed method can detect more and smaller tumor regions.

For instance, show HCH Images and the difference with Camelyons

**Author response:** We very much appreciate the comments. In the revised manuscript, we have made additions in Fig. 6.
